# Measuring physical activity among pregnant women using a structured one-week recall questionnaire: evidence for validity and reliability

**DOI:** 10.1186/1479-5868-7-21

**Published:** 2010-03-21

**Authors:** Kelly R Evenson, Fang Wen

**Affiliations:** 1 Department of Epidemiology, Gillings School of Global Public Health, University of North Carolina, Chapel Hill, North Carolina, USA

## Abstract

**Background:**

Accurate measurement of the components of physical activity during pregnancy can aid in our understanding of the dose response relationships between physical activity and corresponding perinatal outcomes. The aim of this study was to develop and evaluate a one-week recall questionnaire to assess moderate to vigorous physical activity during pregnancy.

**Methods:**

To assess concurrent-related validity, 177 pregnant women (median 18 weeks' gestation, interquartile range (IQR) 15 -23) kept a structured diary and wore an accelerometer (Actigraph) for one week. At the conclusion of the week, they completed the Pregnancy Infection and Nutrition 3 (PIN3) physical activity questionnaire over the telephone. To assess evidence for test-retest reliability, 109 pregnant women (median 19 weeks' gestation, IQR 18-27) completed the questionnaire twice over the telephone, within 48 hours apart, recalling the same two time periods. Spearman correlation coefficients (SCC) and intraclass correlation coefficients (ICC) were used to assess evidence for validity and reliability, respectively.

**Results:**

Comparison of the questionnaire to the structured diary was moderate to substantial (SCC 0.47 to 0.69) for several measures of moderate or vigorous physical activity using either perceived or absolute intensity. Comparison of moderate to vigorous physical activity from the questionnaire (absolute intensity using MET-hours/week) to the accelerometer ranged from 0.12 to 0.23 using SCC for absolute intensity (MET-hours/week) and 0.28 to 0.34 using relative intensity (hours/week) (n = 177). Test-retest reliability was moderate to almost perfect for moderate to vigorous physical activity, with the ICC ranging from 0.56 to 0.82 for both perceived and absolute intensities.

**Conclusions:**

The PIN3 one-week recall questionnaire assessed moderate to vigorous physical activity in the past week with evidence for reliability and validity.

## Background

Several decades of research supports the benefits of physical activity during pregnancy [1,2]. In acknowledgment of this, several physician organizations endorse physical activity during pregnancy with position statements, such as in Canada [3], the United Kingdom [4], and the United States (US) [5]. In addition, the US government included a section on pregnancy recommendations in its national "2008 Physical Activity Guidelines for Americans" [1].

To evaluate and provide more specific evidence towards guidelines, accurate assessment of physical activity during pregnancy is needed. Self-reported questionnaires are useful in this regard, as they are often more economical and provide information on types and perceived intensity of activity not available from objective measurement methods. In epidemiologic studies of pregnant women, one goal for the questionnaire is to properly rank women within a narrow range of physical activity [6].

To date three physical activity questionnaires were evaluated for evidence of both validity and reliability among pregnant women: the Pregnancy Physical Activity Questionnaire [7,8], the modified Kaiser Physical Activity Survey [9], and the modified International Physical Activity Questionnaire [10]. The first two questionnaires [7-9] provide information on the mode, frequency, and duration of physical activity in the current trimester of pregnancy; neither questionnaire collects perceived intensity, but activities can be assigned an absolute intensity through the use of metabolic equivalent (MET) values corresponding to specific modes of activity [11,12]. The third questionnaire is an adaption of the International Physical Activity Questionnaire; it is shorter and provides information on leisure and household activities in the past two weeks [10]. It had low evidence of validity and reliability among a sample of pregnant women. An additional questionnaire that focused only on recreational activity (including active transport) recalled since becoming pregnant was evaluated for evidence of validity; the authors concluded that it may be useful in ranking women according to recreational activity [13]. Three of these questionnaires are self-administered [7,10,13] and one is interviewer-administered [9].

Physical activity during pregnancy may not be stable within a trimester, due to rapid changes in the mother's body. In addition, the compendium of physical activities used to assign MET values to activities is based on adults and does not account for pregnancy. Thus, it may be valuable to collect perceived intensity during the physical activity recall. We developed a one-week recall questionnaire to assess moderate to vigorous physical activity during pregnancy for use in the third Pregnancy Infection and Nutrition (PIN3) Study. It was designed such that dose response could be explored, accounting for variation in physical activity over a shorter time period, and comparisons could be made between perceived (relative) and absolute intensity. The aim of this study was to evaluate evidence for test-retest reliability and concurrent related validity for the PIN3 structured one-week recall physical activity questionnaire among pregnant women.

## Methods

All data collection described herein was approved by the University of North Carolina - Chapel Hill Institutional Review Board and each participant provided written informed consent prior to participation in the study. We evaluated evidence for test-retest reliability and concurrent validity from two independent samples of pregnant women.

### PIN3 Physical Activity Questionnaire

A one-week recall questionnaire was developed to evaluate physical activity among pregnant women enrolled in the PIN3 Study. The questionnaire was interviewer administered and designed to capture moderate and vigorous physical activity. It is available as additional file 1. The questionnaire can be contrasted with an interviewer-administered 7-day recall [14], but instead of recalling day-by-day the participant recalls mode-by-mode. The PIN3 physical activity questionnaire assessed frequency and duration of all moderate and vigorous physical activities the woman participated in, including activity done for recreation, at work, for transportation, childcare, adult care, and both indoor and outdoor household activities. Using recreational activity as an example, the question asked: "In the past week, did you participate in any non-work, recreational activity or exercise, such as walking for exercise, swimming, or dancing, that caused at least some increase in breathing and heart rate?" If the participant responded 'Yes', then the interviewer asked her to list all types of activities, one by one, with the following question: "What type of recreational activities did you do during the past week?" For each activity, the participant reported the number of sessions per week, duration of each session, and the perceived intensity level using the following options: 'fairly light,' 'somewhat hard,' and 'hard or very hard'. These intensity categories corresponded to the Borg scale of perceived exertion [15]. These questions were repeated for work, transportation, child and adult care giving, indoor household, and outdoor household activity.

The scoring of the questionnaire included assessment of intensity of activity in two ways. First, perceived (or relative) intensity using the modified Borg scale [15] to capture the participant's perception of intensity and to derive number of hours in the past week spent in moderate to vigorous physical activity, We classified activities reported as "somewhat hard" as moderate and activities reported as "hard or very hard" as vigorous. Second, absolute intensity classifying activities using published metabolic (MET) tables [11,12] to determine the number of MET-hours per week spent in physical activity. A number of activities reported by women were not listed in the compendium. Thus, two raters determined the MET intensity based on other activities in the compendium; when the two raters disagreed they met and resolved by consensus. The final compendium of activities used for scoring is available elsewhere http://www.cpc.unc.edu/projects/pin/design_pin3/docs_3/PIN-MET-Table-080207.pdf. To determine moderate activity using absolute intensity, we defined moderate intensity as both 3 to 6 METS, a definition often used for adults [16], and 4.8 to 7.1 METS, the estimated values for moderate activity specific to adults 20 to 39 years of age [17].

The questionnaire took on average 10-20 minutes to administer. Intra- and inter-interviewer quality control measures, such as expert review of taped interviews, were established to ensure that interviewers were asking questions reliably and systematically.

### Concurrent-Related Validity Assessment

For this portion of the study, pregnant women from central North Carolina were recruited by placing flyers in local clinics and screened over the telephone. Women were not enrolled if they were non-English speaking, under the age of 18 years, carrying multiple gestations, did not have a telephone from which they could complete the phone interviews, or more than 28 weeks' gestation during the screening call. The women wore an accelerometer for one week, kept a daily structured diary, and following this completed a questionnaire that included the one-week recall of the PIN3 physical activity questionnaire. Participants were paid $50 at the completion of the study; later in the study an electromagnetic field monitor was added to the protocol [18] and participants were paid $75.

#### PIN3 Diary Card

The structured diary card was developed as a way of assessing the evidence for concurrent related validity of the PIN3 physical activity questionnaire. The card and the instructions can be found in additional file 2. The goal of the card was to collect all moderate to vigorous physical activities the women performed in the past week. Women were requested to fill out the two-page card on a daily basis and to mail it back to us at the end of the one week period. The activities listed were the ones most commonly reported among the initial PIN3 participants and space was provided to list other types of activities. For each day, participants filled in time spent and perceived exertion (based on the same intensity descriptions used in the questionnaire) for each activity. On the diary card, if a woman performed an activity at two different intensities, such as gardening, one intensity level and time could be recorded in one row and the activity along with the different intensity and time could be recorded as an "other activity". In practice, this did not happen and so we assume women recorded the intensity level that best represented that activity.

The scoring of the diary card was similar as possible to the scoring of the PIN3 questionnaire. Hours per week in physical activity was calculated by adding up the time reported for each specific activity by intensity level. All activities were assigned a MET value from the compendium [11,12], in order to determine MET-hours of activity.

#### Accelerometer

For the accelerometer, we used the Manufacturing Technology Inc. (MTI) Actigraph accelerometer model #7164 (Fort Walton Beach, FL). It is a small, light-weight uniaxial accelerometer that measures accelerations in the range of 0.05 to 2 G's with a band limited frequency of 0.25-2.5 Hertz [19]. Validity [20-22] and reliability [23-26] of the monitor as an indicator for physical activity have been demonstrated among adults.

Women were fitted with the accelerometer to be worn during waking hours for 7 days on a belt or clip-on pouch over their right hip at the iliac crest. They were asked to remove the monitor for sleeping, bathing, or swimming. Written and verbal instructions, as well as a phone number to call with questions, were provided. Participants mailed the monitor back to the study offices at the conclusion of the 7 days.

Accelerometer data were collected with 1-minute epochs, and the monitors were regularly calibrated throughout the study. Spurious counts were flagged, assessed, and set to missing if determined to be invalid. We defined non-wear time as a period of 20 minutes or more of zeros. We defined a standard measurement day as the length of time in which > = 70% of the sample was wearing the accelerometer, separately for weekdays and weekends similar to others [27]. We classified participants as having complete accelerometry data if they had nonmissing counts over at least 70% of a standard measurement day for all 7 days.

Ignoring missing values in the accelerometer file can cause a biased estimate of the true level of physical activity [27]. Therefore, missing data were filled by using multiple imputation inference strategy (using SAS proc MI) through expectation-maximization algorithm and a Markov Chain Monte Carlo method [27]. Considering the wearing time of our participants, non-missing accelerometer data falling into the daily time window of 5 am to midnight was selected as reference data for the imputation. Indicators on ten blocks of the time period (5 am to midnight) and week day versus weekend were used for the imputation procedure. In order to represent a random sample of the missing values, ten multiple imputed data sets were created by the multiple imputation procedure and each imputation contained minute-by-minute daily activity count data from 5 am to midnight.

From the accelerometer, we used the data several ways. First, using total counts per week we evaluated the raw data provided by the accelerometer without imposing cutpoint decisions. Second, activity was calculated as hours per week (using count thresholds) and counts per week spent in differing intensities (e.g., light, moderate, vigorous). A number of calibration studies of adults provide count thresholds (e.g., cutpoints) for moderate and vigorous activity. We calculated cutpoints using three of these studies: Freedson et al [20], Swartz et al [28], and summary cutpoints from National Heath and Nutrition Examination Survey (NHANES) data [29], calculated originally by taking the weighted average of cutpoints from Freedson et al [20], Yngve et al [30], Leenders et al [31], and Brage et al [22].

### Test-Retest Reliability Assessment

For this portion of the study, we relied on a sample of PIN3 participants. The PIN3 Study recruited pregnant women less than 20 weeks' gestation seeking prenatal care at clinics associated with the University of North Carolina Hospitals. Trained staff identified women through review of all medical charts of new prenatal patients. Women were not enrolled if they were non-English speaking, under the age of 16 years, carrying multiple gestations, not planning to continue care or deliver at the study hospital, or did not have a telephone from which they could complete the phone interviews. Selected women were asked to participate in two telephone interviews, at 17-22 and 27-30 weeks' gestation. The study website http://www.cpc.unc.edu/pin provides greater detail on the protocols and measures.

We evaluated the physical activity questionnaire test-retest reliability among pregnant women within 48 hours of their telephone interview completion as part of the PIN3 Study. We enrolled women purposefully, with approximately equal numbers in six strata: (i) completing the first (17-22 weeks) or second (27-30 weeks) telephone interview and (ii) self-report of either being currently employed in a strenuous job, active at leisure, or inactive at either work or leisure. During the second interview, women were asked to recall the same week that the first interview was conducted, so that recall periods matched. Women were paid $5 for participation in each interview.

### Other Measures

During the first interview for both the validity and reliability samples, women were asked to report their education, current work status, race, general health, and parity (live plus still births). For the reliability sample only, age was reported at the interview and the medical record was abstracted and included self-reported weight and measured height for the determination of pre-pregnancy body mass index (BMI). BMI values were grouped using the Institute of Medicine recommendations for pregnant women, in effect during that time period, into low (<19.8 kg/m^2^) or normal weight (19.8-<26.0 kg/m^2^), overweight (26.0-<30.0 kg/m^2^), and obese (> = 30.0 kg/m^2^) [32]. For the validity samples, age was obtained during recruitment and calculated based on her date of birth as her age on the interview date.

### Statistical Analysis

All analyses were conducted using SAS version 9.1 (Cary, NC). Evidence for validity was assessed using Spearman correlation coefficients (SCC) with 95% confidence intervals (CI) comparing either the diary or accelerometer results to the questionnaire. We conducted analyses comparing the accelerometer to the questionnaire using the full sample, the imputed sample, and the smaller sample defined by complete accelerometry data. Bland-Altman plots [33] were used to assess agreement between physical activity measurements from the questionnaire with either the accelerometer or the diary among the full sample. On the plots, the x-axis is the average of two measurements, while the y-axis is the difference between the two measurements. Horizontal lines represent the upper and lower limits of agreement and the mean difference of the two measurements. In addition, Pitman's test of differences in the variances was calculated for each comparison [34].

Test-retest reliability was assessed using intraclass correlations coefficients (ICC) with corresponding 95% CI. ICC were calculated using a two-way analysis of variance model and conceptualized as the proportion of the total variance explained by between-participant variance. Bland-Altman plots [33] were used to compare questionnaire results at the two time periods. Stratified analyses were performed to determine if differences in reliability differed by gestational age at interview date, race, education, age, parity, or pre-pregnancy BMI. As a rough guide, we followed the ratings suggested by Landis and Koch [35] for agreement level: 0-0.20 slight, 0.21-0.40 fair, 0.41-<0.60 moderate, 0.61-<0.80 substantial, and 0.81-<1.00 almost perfect. Each physical activity entry was screened and outliers were pairwise deleted from the analysis.

## Results

### Description of Participants

In total, 177 pregnant women participated in the validity study and 109 pregnant women participated in the reliability study. For the validity study, a standard measurement day in which 70% of the women were wearing the accelerometer was defined from 9:00 to 21:00 on weekdays and 10:30 to 21:00 on weekends (see Figure in additional file 3). Thus, the minimum number of hours of nonmissing data allowed to be considered as having complete accelerometry data was 8.4 hours on weekdays (12 hours * 70%) and 7.4 hours on weekends (10.5 hours * 70%). Among our sample, 120 women (68%) met this criteria and were defined as the "complete Actigraph sample".

A description of participants in the validity (separately for the full sample and a subset of those determined to have worn the accelerometer for the minimum defined time over 7 days) and reliability studies are reported in Table 1. The median gestational week that the validity sample participated was 18 weeks (n = 177, interquartile range 15 to 23 weeks) and the reliability sample participated at 19 weeks (n = 109, interquartile range 18 to 27 weeks). The validity sample (n = 177) included a lower proportion of Black women, those with less than or equal to a high school education, younger age groups (<20 years), and lower general health (combining good, fair, and poor categories) compared to the reliability sample. The work status between the groups was similar. The validity sample reported more moderate, vigorous, and moderate to vigorous physical activity than the reliability sample (see Table in additional file 4).

**Table 1:** Descriptive information on the participants in the validity and reliability studies#

	Validity Study				Reliability Study	
		
	Complete Actigraph Sample*		Full Sample			
			
	n = 120	%	n = 177	%	n = 109	%
Race						
White	87	72.5	119	67.2	79	72.5
Black	17	14.2	38	21.5	29	26.6
Other	16	13.3	20	11.3	1	0.9
						
Education						
< = High school	1	0.8	8	4.5	35	33.7
Some college	22	18.3	40	22.6	14	13.5
College +	97	80.8	129	72.9	55	52.9
						
Age group						
<20	2	1.7	7	4.0	9	8.3
20-29	45	37.5	76	43.0	47	43.1
30+	73	60.8	94	53.1	53	48.6
						
General Health						
Excellent	60	50.0	74	41.8	34	31.2
Very Good	44	36.7	74	41.8	46	42.2
Good	12	10.0	22	12.4	22	20.2
Fair	2	1.7	5	2.8	7	6.4
Poor	2	1.7	2	1.1	0	0.0
						
Currently working						
Yes	78	65.0	118	66.7	72	66.1
No	42	35.0	59	33.3	37	33.9

*Complete data was defined as having nonmissing counts over at least 70% of a standard measurement day, with a standard measurement day defined as the length of time in which at least 70% of participants were wearing the monitor. This was defined separately for weekdays and weekends.

#In some cases the values may not add to the sample size due to missing values.

### Concurrent-Related Validity Assessment Using a Diary

To assess evidence for validity of the questionnaire, we compared summary values from the structured PIN3 diary to the physical activity questionnaire, with the average values from the diary reported in additional file 4. Agreement using SCC was moderate to substantial (0.47 to 0.69) for moderate, vigorous, moderate to vigorous, and total physical activity assessed using either perceived or absolute intensity (Table 2). Results from the Bland-Altman plots (not shown) indicated that the results from the diary were more often higher than the results from the questionnaire for total hours/week (85.9% of women from the full sample), total MET-hours/week (82.5%), moderate to vigorous hours/week (67.8%), and moderate to vigorous MET-hours/week using 3-6 METS to define moderate activity (80.2%). Of these four measures, only moderate to vigorous physical activity included exact matches (7.3% of the sample). For moderate to vigorous MET-hours/week using 4.8-7.1 METS to define moderate activity, the diary consistently provided similar measures as the questionnaire (47.5% having the same measure) and a similar distribution of over and under reporting as compared to the questionnaire (24.3%, 28.2%, respectively). The Pitman's test of differences in the variances supported these observations, with only the absolute measure of moderate to vigorous MET-hours/week (using 4.8-7.1 METS to define moderate activity) showing no significant differences in variances.

**Table 2:** Comparison of the PIN3 physical activity questionnaire to the diary card using Spearman correlation coefficients (SCC) with 95% confidence intervals (CI)

Comparison Variables*	Diary Card (n = 177) SCC (95% CI)
Questionnaire using perceived intensity (hours/week)	
Total physical activity	0.67 (0.57, 0.76)
Moderate activity	0.63 (0.52, 0.73)
Vigorous activity	0.68 (0.56, 0.79)
Moderate to vigorous activity	0.66 (0.56, 0.76)
	
Questionnaire using absolute intensity (MET-hours/week)	
Total physical activity	0.63 (0.53, 0.73)
	
Moderate intensity defined as 3-6 METS	
Moderate activity	0.62 (0.52, 0.73)
Vigorous activity	0.53 (0.39, 0.67)
Moderate to vigorous activity	0.60 (0.49, 0.70)
	
Moderate intensity defined as 4.8-7.1 METS	
Moderate activity	0.61 (0.49, 0.74)
Vigorous activity	0.47 (0.29, 0.64)
Moderate to vigorous activity	0.69 (0.58, 0.80)

* The diary card and questionnaire variables compared to each other were intended to assess the same intensities of physical activity.

### Concurrent-Related Validity Assessment Using an Accelerometer

Evidence for validity was also assessed by comparing accelerometer measures collected during the same week as the recall period for the physical activity questionnaire, with the average values from the accelerometer reported in additional file 4. Table 3 reports findings using absolute intensity measures from the questionnaire. The results were generally similar whether we used the full sample (n = 177), the sample that included imputation (n = 177), or the sample with complete Actigraph data over 7 days (n = 120). SCC were fair, ranging from 0.20 to 0.31 when comparing total Actigraph counts/week to the questionnaire in MET-hours/week. Results were generally similar whether we defined moderate intensity as 3 to 6 METS or 4.8 to 7.1 METS. Considering the lower count cutpoints using the Swartz et al calibration study results, moderate activity agreement ranged from 0.04 to 0.24, while vigorous activity ranged from 0.29 to 0.44, and total moderate to vigorous activity ranged from 0.10 to 0.32. Considering the higher count cutpoints using the Freedson et al and Troiano et al cutpoints for either moderate activity definition, agreement ranged from 0.01 to 0.07, while vigorous activity ranged from 0.28 to 0.40, and total moderate to vigorous activity agreement ranged from 0.12 to 0.20.

**Table 3:** Comparison of the PIN3 physical activity questionnaire to the accelerometer using Spearman correlation coefficients (SCC) with 95% confidence intervals (CI), using absolute intensity overall and separately using 3-6 METS and 4.8-7.1 METS to define moderate activity

	Accelerometry in Counts/Week Using Various Cutpoints*			Total Counts/Week
		
	Freedson et al	Swartz et al	Troiano et al	
	
	SCC (95% CI)	SCC (95% CI)	SCC (95% CI)	SCC (95% CI)
Total physical activity in MET-hours/week				
Full sample**				0.23 (0.08, 0.38)
Complete Actigraph sample***				0.31 (0.13, 0.49)
Imputed data sample#				0.20 (0.05, 0.35)
				
Physical activity in MET-hours/week, using 3-6 METS to define moderate activity				
Moderate activities				
Full sample**	0.01 (-0.13, 0.16)	0.14 (-0.02, 0.29)	0.02 (-0.12, 0.16)	
Complete Actigraph sample***	0.07 (-0.11, 0.24)	0.24 (0.05, 0.43)	0.06 (-0.12, 0.24)	
Imputed data sample#	0.03 (-0.12, 0.17)	0.18 (0.03, 0.33)	0.02 (-0.12, 0.16)	
Vigorous activities				
Full sample**	0.34 (0.18, 0.49)	0.42 (0.28, 0.55)	0.34 (0.19, 0.50)	
Complete Actigraph sample***	0.38 (0.20, 0.56)	0.44 (0.28, 0.60)	0.40 (0.22, 0.57)	
Imputed data sample#	0.33 (0.17, 0.48)	0.41 (0.27, 0.54)	0.33 (0.18, 0.49)	
Moderate to vigorous activity				
Full sample**	0.13 (-0.02, 0.27)	0.23 (0.09, 0.38)	0.12 (-0.02, 0.27)	0.24 (0.10, 0.39)
Complete Actigraph sample***	0.17 (0.004, 0.35)	0.32 (0.14, 0.50)	0.16 (-0.01, 0.34)	0.32 (0.14, 0.50)
Imputed data sample#	0.13 (-0.01, 0.27)	0.21 (0.06, 0.35)	0.12 (-0.02, 0.27)	0.22 (0.07, 0.36)
				
Physical activity in MET-hours/week, using 4.8-7.1 METS to define moderate activity				
Moderate activities				
Full sample**	0.05 (-0.10, 0.20)	0.08 (-0.07, 0.23)	0.04 (-0.11, 0.19)	
Complete Actigraph sample***	0.05 (-0.14, 0.24)	0.05 (-0.13, 0.23)	0.04 (-0.15, 0.22)	
Imputed data sample#	0.04 (-0.12, 0.19)	0.04 (-0.11, 0.18)	0.03 (-0.12, 0.18)	
Vigorous activities				
Full sample**	0.29 (0.13, 0.45)	0.30 (0.15, 0.46)	0.31 (0.15, 0.47)	
Complete Actigraph sample***	0.32 (0.14, 0.51)	0.32 (0.14, 0.50)	0.34 (0.15, 0.52)	
Imputed data sample#	0.28 (0.12, 0.44)	0.29 (0.14, 0.44)	0.30 (0.14, 0.46)	
Moderate to vigorous activity				
Full sample**	0.19 (0.04, 0.33)	0.21 (0.06, 0.35)	0.18 (0.04, 0.33)	0.22 (0.07, 0.36)
Complete Actigraph sample***	0.20 (0.02, 0.38)	0.21 (0.04, 0.38)	0.19 (0.01, 0.37)	0.21 (0.04, 0.38)
Imputed data sample#	0.15 (0.002, 0.31)	0.10 (-0.05, 0.24)	0.16 (0.01, 0.31)	0.09 (-0.05, 0.24)

*The cutpoints on accelerometer counts per minute were defined as follows:

Freedson et al: 1952-5724 (Moderate); > = 5725 (Vigorous)

Troiano et al: 2020-5998 (Moderate); > = 5999 (Vigorous)

Swartz et al: 574-4944 (Moderate); > = 4945 (Vigorous)

**Full sample n = 177

***Complete Actigraph data (n = 120) was defined as having nonmissing counts over at least 70% of a standard measurement day, with a standard measurement day defined as the length of time in which at least 70% of participants were wearing the monitor. This was defined separately for weekdays and weekends.

#Imputed sample n = 177

Similar results were found when scoring the questionnaire using perceived intensity in hours/week (Table 4). Agreement results were generally similar using any of the three cutpoints, and either the full sample (n = 177) or the sample with complete Actigraph data over 7 days (n = 120). Agreement between moderate activity on the accelerometer and the questionnaire ranged from 0.16 to 0.33, vigorous activity agreement ranged from 0.26 to 0.33, and moderate to vigorous activity agreement ranged from 0.22 to 0.34. Results from the Bland-Altman plots (not shown) indicated that moderate to vigorous physical activities reported on the questionnaire (hours/week) were higher when compared to those derived from the accelerometer using the Freedson or Troiano cutpoints. However, using the Swartz et al cutpoints, higher hours/week of moderate to vigorous physical activity from the accelerometer was observed in 94.4% of the sample compared to the questionnaire. Pitman's test of difference in variance supported the trends from Bland-Altman plots (not shown) and indicated that there were significant differences between the activity measures from accelerometer and reported on the questionnaire.

**Table 4:** Comparison of the PIN3 physical activity questionnaire to the accelerometer using Spearman correlation coefficients (SCC) with 95% confidence intervals (CI), using perceived intensity

	Accelerometry in hours/week using cutpoints *		
	
	Freedson et al	Swartz et al	Troiano et al
	
	SCC (95% CI)	SCC (95% CI)	SCC (95% CI)
Questionnaire (hours/week)			
Moderate activity			
Full sample**	0.25 (0.11, 0.39)	0.33 (0.18, 0.47)	0.25 (0.11, 0.39)
Complete Actigraph sample***	0.16 (-0.01, 0.34)	0.25 (0.08, 0.43)	0.16 (-0.01, 0.34)
Imputed data sample#	0.20 (0.06, 0.34)	-0.03 (-0.19, 0.12)	0.20 (0.06, 0.35)
Vigorous activity			
Full sample**	0.29 (0.13, 0.45)	0.26 (0.11, 0.42)	0.32 (0.16, 0.47)
Complete Actigraph sample***	0.32 (0.13, 0.51)	0.32 (0.13, 0.51)	0.33 (0.14, 0.52)
Imputed data sample#	0.29 (0.14, 0.45)	0.27 (0.11, 0.42)	0.32 (0.16, 0.48)
Moderate to vigorous activity			
Full sample**	0.29 (0.15, 0.42)	0.34 (0.20, 0.48)	0.28 (0.14, 0.41)
Complete Actigraph sample***	0.23 (0.06, 0.40)	0.30 (0.13, 0.47)	0.22 (0.05, 0.39)
Imputed data sample#	0.24 (0.10, 0.37)	0.002 (-0.15, 0.16)	0.23 (0.10, 0.37)

*The cutpoints on accelerometer counts per minute were defined as follows:

Freedson et al: 1952-5724 (Moderate); > = 5725 (Vigorous)

Swartz et al: 574-4944 (Moderate); > = 4945 (Vigorous)

Troiano et al: 2020-5998 (Moderate); > = 5999 (Vigorous)

**Full sample n = 177

***Complete Actigraph data (n = 120) was defined as having nonmissing counts over at least 70% of a standard measurement day, with a standard measurement day defined as the length of time in which at least 70% of participants were wearing the monitor. This was defined separately for weekdays and weekends.

#Imputed sample n = 177

### Test-Retest Reliability Assessment

Test-retest was moderate to almost perfect for total and moderate to vigorous physical activity, with the ICC ranging from 0.56 to 0.84 for both perceived and absolute intensities (Table 5). When considering moderate to vigorous activity, reliability for perceived hours/week in "somewhat hard" activity was lower (0.56, 95% CI 0.42, 0.68) than for absolute MET-hours/week for either the lower definition of 3-6 METS (0.82, 95% CI 0.75, 0.87) or the higher definition of 4.8-7.1 METS (0.74, 95% CI 0.64, 0.82). Results from the Bland-Altman plots (not shown) indicated an even distribution of values above and below the difference line of zero, with more discrepant findings occurring only at the highest reported mean values of physical activity, using either perceived or absolute total activity or moderate to vigorous physical activity.

**Table 5:** Test-retest reliability of PIN3 physical activity questionnaire using intraclass correlation coefficients (ICC) with 95% confidence intervals (CI) (n = 109)

	Test retest reliability
	**ICC (95% CI)**
Overall:	
hours/week using perceived intensity	
Total	0.84 (0.78, 0.89)
Moderate to vigorous	0.56 (0.42, 0.68)
Moderate	0.54 (0.39, 0.66)
Vigorous	0.40 (0.23, 0.55)
MET-hours/week using absolute intensity	
Total	0.84 (0.77, 0.89)
Moderate to vigorous (MET> = 3)	0.82 (0.75, 0.87)
Moderate	0.82 (0.75, 0.88)
Vigorous	0.75 (0.66, 0.82)
Moderate to vigorous (MET> = 4.8)	0.74 (0.64, 0.82)
Moderate	0.72 (0.61, 0.80)
Vigorous	0.73 (0.63, 0.81)
	
By Mode of Physical Activity:	
Work activity:	
hours/week using perceived intensity	
Total	0.56 (0.41, 0.67)
Moderate to vigorous	0.75 (0.65, 0.82)
MET-hours/week using absolute intensity	
Total	0.59 (0.46, 0.70)
Moderate to vigorous (MET> = 3)	0.59 (0.45, 0.70)
Moderate to vigorous (MET> = 4.8)	0.60 (0.47, 0.71)
Recreational activity:	
hours/week using perceived intensity	
Total	0.86 (0.80, 0.90)
Moderate to vigorous	0.89 (0.84, 0.92)
MET-hours/week using absolute intensity	
Total	0.85 (0.79, 0.90)
Moderate to vigorous (MET> = 3)	0.83 (0.77, 0.88)
Moderate to vigorous (MET> = 4.8)	0.81 (0.73, 0.86)
	
Outdoor household activity:	
hours/week using perceived intensity	
Total	0.91 (0.88, 0.94)
Moderate to vigorous	0.76 (0.67, 0.83)
MET-hours/week using absolute intensity	
Total	0.89 (0.85, 0.93)
Moderate to vigorous (MET> = 3)	0.88 (0.83, 0.92)
Moderate to vigorous (MET> = 4.8)	0.80 (0.72, 0.86)
Indoor household activity:	
hours/week using perceived intensity	
Total	0.67 (0.55, 0.76)
Moderate to vigorous	0.39 (0.22, 0.54)
MET-hours/week using absolute intensity	
Total	0.58 (0.45, 0.70)
Moderate to vigorous (MET> = 3)	0.36 (0.18, 0.51)
Moderate to vigorous (MET> = 4.8)	0.62 (0.49, 0.72)
	
Child and adult care activity:	
hours/week using perceived intensity	
Total	0.75 (0.65, 0.82)
Moderate to vigorous	0.51 (0.36, 0.64)
MET-hours/week using absolute intensity	
Total	0.75 (0.65, 0.82)
Moderate to vigorous (MET> = 3)	0.75 (0.65, 0.82)
Moderate to vigorous (MET> = 4.8)	0.96 (0.94, 0.97)
	
Transportation activity:	
hours/week using perceived intensity	
Total	0.77 (0.68, 0.83)
Moderate to vigorous	0.68 (0.57, 0.77)
MET-hours/week using absolute intensity	
Total	0.77 (0.68, 0.83)
Moderate to vigorous (MET> = 3)	0.77 (0.68, 0.83)
Moderate to vigorous (MET> = 4.8)	NA

NA = not applicable because no transportation activities were > = 4.8 METS

Test-retest reliability remained moderate to almost perfect, using either perceived or absolute intensity, when exploring by modality for work, recreation, outdoor household, child and adult care, and transportation activity (Table 5). The exception to this was moderate to vigorous indoor household activity, where the ICC was 0.39 for perceived intensity and 0.36 for absolute intensity using a 3 MET lower bound.

Next we explored whether test-retest reliability differed by several factors, according to the ICC, for total moderate to vigorous physical activity using both perceived and absolute intensity definitions (Table 6). The factors included interview date (17-22 or 27-30 weeks' gestation), race (White or Other), education (high school or less, some college or more), age (18 to 29 years, 30 or more years), parity (zero or 1+), and prepregnancy body mass index (<25 or > = 25 kg/m^2^). Only a few meaningful differences were identified, defined as a difference that would classify women into a different category as described by Landis and Koch [35]. For total moderate to vigorous hours/week, White women and younger women had moderate reliability while women of other races and older women had substantial reliability. Furthermore, women with lower education had fair reliability on this measure, while those with higher education had substantial reliability. The only difference identified for total moderate to vigorous MET-hours/week defined at > = 3 METS was for race; White women had almost perfect reliability while women of other races had substantial reliability. For total moderate to vigorous MET-hours/week defined at > = 4.8 METS, those with lower education had moderate reliability while those with higher education had substantial reliability and those who were younger had fair reliability while those that were older had almost perfect reliability.

**Table 6:** Stratified test-retest reliability of PIN3 physical activity questionnaire using intraclass correlation coefficients (ICC) with 95% confidence intervals (CI) (n = 109*)

		Total moderate to vigorous hours/week (perceived exertion)	Total moderate to vigorous MET-hours/week (> = 3 METS)	Total moderate to vigorous MET-hours/week (> = 4.8 METS)
				
	N	ICC (95% CI)	ICC (95% CI)	ICC (95% CI)
Interview Dates:				
Time 1 (17-22 weeks' gestation)	66	0.64 (0.47, 0.76)	0.81 (0.70, 0.88)	0.71 (0.57, 0.81)
Time 2 (27-30 weeks' gestation)	43	0.49 (0.23, 0.69)	0.84 (0.72, 0.91)	0.81 (0.68, 0.89)
				
Race:				
White	79	0.51 (0.32, 0.65)	0.87 (0.80, 0.91)	0.73 (0.61, 0.82)
Other	30	0.73 (0.52, 0.86)	0.65 (0.39, 0.82)	0.74 (0.53, 0.87)
				
Education:				
High school or less	49	0.34 (0.01, 0.60)	0.80 (0.64, 0.89)	0.51 (0.22, 0.72)
Some college or above	60	0.71 (0.57, 0.80)	0.82 (0.74, 0.89)	0.75 (0.64, 0.84)
				
Age:				
18-29 years	56	0.45 (0.21, 0.64)	0.87 (0.79, 0.92)	0.35 (0.08, 0.56)
30 or more years	53	0.68 (0.47, 0.81)	0.88 (0.79, 0.93)	0.82 (0.68, 0.90)
				
Parity:				
Zero	53	0.58 (0.37, 0.74)	0.66 (0.47, 0.79)	0.73 (0.57, 0.83)
1 or more	56	0.56 (0.35, 0.71)	0.83 (0.73, 0.90)	0.76 (0.62, 0.85)
				
Prepregnancy BMI:				
<25 kg/meters squared	63	0.51 (0.31, 0.67)	0.82 (0.72, 0.89)	0.80 (0.69, 0.87)
25 or more kg/meters squared	46	0.69 (0.50, 0.82)	0.79 (0.66, 0.88)	0.62 (0.40, 0.77)

*In some cases the sample sizes may not add to 109 due to missing values.

## Discussion

In epidemiologic studies, physical activity questionnaires should rank individuals from sedentary to most active; the challenge may be greater among pregnant women who may not be as active [36] (and thus the distribution of their activity is less) as their non-pregnant counterparts [6]. We developed a one-week recall instrument, to obtain more frequent fluxuations in physical activity that may occur during pregnancy, and evaluated the psychometric properties of the questionnaire. These results indicate that the questionnaire generally displayed evidence for fair to substantial concurrent validity and moderate to substantial test-retest reliability.

### Evidence for Validity Comparing to the Diary

With the lack of a single comparison standard measure for physical activity, we used both a self-reported (diary) and objective (accelerometer) measure to compare to the physical activity questionnaire. The PIN3 structured diary provided estimates of moderate to vigorous physical activity over the same week that the questionnaire recall occurred. SCC were moderate to substantial regardless of whether perceived or absolute intensity from the questionnaire was used. While there is some concern that keeping the structured diary may heighten participant's awareness and report of their recalled physical activities on the questionnaire for the validation portion of the study, thus biasing results to appear more favorable than they are, others have shown that this does not affect overall estimates of validity in other populations [37].

### Evidence for Validity Comparing to the Accelerometer

A concern when comparing two self-reported measures against one another is that the errors inherent with each method may be correlated, resulting in inflated estimates of validity [9]. To account for this, we also compared the PIN3 physical activity questionnaire to an objective measure of physical activity. We found that agreement was higher when comparing the questionnaire to the diary (i.e., both self-reported measures) in comparison to the accelerometer.

For accelerometry, the challenge is that the cutpoints used to convert counts to moderate and vigorous intensity activity vary across studies, due in part to different calibration methods, different activities included in the calibration study, and differing populations. For adults, there seems to be more variability with moderate activity cutpoints as compared to vigorous activity cutpoints and no calibration studies have been conducted on pregnant women. We explored the sensitivity of the objective validity results by exploring three different accelerometry cutpoints for moderate and vigorous physical activity. The cutpoints from Freedson et al [20] and Swartz et al [28] were used in other pregnancy studies collecting accelerometry data [7,9]. In addition, we used summary cutpoints from NHANES data by Troiano et al [29]. Similar to Chasan-Taber et al [7], we found that the correlation between the accelerometer and the questionnaire were lower for the Freedson et al [20] equation in which the count threshold was much higher, than for equations using lower thresholds from Swartz et al [28]. Others have also found that the higher count cutpoints for moderate activity may be set too high to capture a broad range of moderate activities [38]. Therefore, in addition to reporting comparisons using these cutpoints, we reported correlations with Actigraph counts, an indicator of overall physical activity that does not rely on thresholds.

We also explored our validation results using cutpoints for moderate to vigorous physical activity from a calibration study of Hendelman et al [21], which assigned moderate activity at 191 counts/minute and vigorous activity at 7525 counts/minute. This cutpoint for moderate activity was explored in other studies of pregnancy [7,9,39] and is much lower than the other values that we explored, while the vigorous cutpoint is higher than any we used. While the correlational results using Hendelman cutpoints generally produced similar results to our findings, the Bland-Altman plots indicated a strong bias with almost all women having higher values from the accelerometer as compared to the questionnaire. Thus, we chose not to report these results.

While the accelerometer is an acceptable measure to compare a physical activity measure against due to its objectivity and removal of recall and response bias, it is not without limitations. The accelerometer we used (Actigraph) is uniaxial; thus activities that involved upper body movement, lifting, or cycling are underestimated. Moreover, swimming activities were not counted, since we asked women to remove the accelerometer when in the water.

A recent comprehensive review summarized the results of studies comparing physical activity questionnaire data to objective measures [40]. When considering comparison to accelerometry, the average percent mean difference (calculated as (self-reported mean minus accelerometer mean)/accelerometer mean) across 60 studies of women was 138%. Most often, women's report of physical activity was higher than the accelerometer readings. In our study, this depended on the validity measure used. On the full sample, the percent mean difference ((mean from self-report - mean from accelerometer)/mean from accelerometer) for moderate to vigorous physical activity was 85% using the Freedson et al cutpoint and 96% using the Troiano et al cutpoint, indicating higher reporting from the questionnaire. In contrast, the percent mean difference for moderate to vigorous physical activity using the Swartz et al cutpoint was -69%, indicating higher reporting from the accelerometer. Using total counts from the accelerometer compared to total physical activity from the questionnaire, the percent mean difference was -88%.

Using the Troiano et al cutpoint, the US national data from 2003 to 2004 indicate that women 20 to 29 years of age average 24 minutes/day of moderate to vigorous physical activity, whereas women 30 to 39 years of age averaged 21 minutes/day [29]. Interestingly, when using the same accelerometer cutpoint we obtained a slightly lower average of 19 minutes/day of moderate to vigorous physical activity among our sample of 177 pregnant women. In contrast, the PIN3 questionnaire averaged 37 minutes/day for these same 177 pregnant women, a two-fold higher report.

### Evidence for Reliability

Test-retest reliability agreement for the PIN3 questionnaire was moderate to almost perfect for moderate to vigorous physical activity. This is similar to reliability assessed from two other physical activity questionnaires, indicating agreement in measuring total physical activity with an ICC of 0.78 [7] and 0.84 [9]. Because we over sampled women who were active, our results may be an underestimate, since test-retest reliability is generally higher among sedentary adults. The period of recall was 24 to 48 hours, in order for women to recall the same time period and not introduce bias in reporting of different days. This short time period between interviews may have influenced their recall of activities, thus leading to higher estimates of reliability.

### Limitations

Several limitations of this study should be noted. First, the distribution of characteristics of women in the validity and reliability samples showed were somewhat different due to the differing methods of recruitment. This would become important if the findings differed by important confounding variables. Second, it is not known how representative our sample is to the source population or how generalizable our results will be to other populations. Comparing to the US national data from NHANES described earlier, among women 20 to 39 years of age, the minutes of moderate to vigorous physical activity was slightly lower among our sample, which seemed reasonable since the women were pregnant. Third, we assigned MET values to all reported activities based on the compendium of physical activities for adults [11,12]. It is not known how well these MET values correspond to similar activities performed while pregnant, but others indicate that they can differ from 0.2 to 2.0 METS [7]. The correlation between physical activity from self-report compared to accelerometry may decline over the course of pregnancy [39]. Studies assessing the metabolic intensity of activities among pregnant women at various gestational ages are needed to help quantify this error. Fourth, we have tested many associations but did not adjust for multiple testing. Therefore, significance should be interpreted with caution, and replication of results would be useful. Lastly, women were asked to recall moderate and vigorous activities that caused at least some increase in their breathing and heart rate. The activities that we classified as less than moderate, with a perceived intensity of "fairly light", may be under ascertained by some women since they were instructed to recall moderate to vigorous activities.

## Conclusion

The PIN3 physical activity questionnaire was designed to provide the frequency, intensity, duration, and mode of physical activities in the past week. The questionnaire is interviewer-administered and asks women to recall moderate to vigorous physical activities mode by mode. Using both direct and indirect methods for evaluation, this study provides validity and reliability evidence for use of the PIN3 questionnaire to assess moderate to vigorous physical activity in the past week among pregnant women.

## Competing interests

The authors declare that they have no competing interests.

## Authors' contributions

KRE conceptualized the project, designed the questionnaires, provided input on data collection, and drafted the manuscript. FW conducted most of the statistical analyses and provided feedback on several earlier drafts of the manuscript. Both authors read and approved the final manuscript.

## Supplementary Material

Additional file 1 **PIN3 physical activity questionnaire, using a structured one-week recall of moderate to vigorous physical activity for pregnant women**. This file provides a summary of the PIN3 physical activity questionnaire that is being evaluated in this study. Click here for file

Additional file 2 **PIN3 physical activity diary card**. This file provides the diary card used for the validation component in this study, along with a scoring table. Click here for file

Additional file 3 **Cumulative proportion of the validity participants (n = 177) wearing the accelerometer by time of day, separately for weekdays and weekends**. This file provides a summary of the accelerometer wearing time among the pregnant women who participated in the validity study. Click here for file

Additional file 4 **Physical activity for the validity and reliability study participants**. This file provides a summary of the physical activity among pregnant women who participated in the validity or reliability study. Click here for file
