# Supplementary material for: Measuring physical activity among pregnant women using a structured one-week recall questionnaire: evidence for validity and reliability
Source: Int J Behav Nutr Phys Act. 2010 Mar 21;7:21. doi: 10.1186/1479-5868-7-21 (PMC2855515; doi:10.1186/1479-5868-7-21)
Supplement: Additional file 1 — PIN3 physical activity questionnaire, using a structured one-week recall of moderate to vigorous physical activity for pregnant women. This file provides a summary of the PIN3 physical activity questionnaire that is being evaluated in this study. [file 1479-5868-7-21-S1.PDF]

Additional File 1: PIN3 physical activity questionnaire, using a structured one-week recall of moderate to vigorous physical activity for pregnant women

	Question	Type	Frequency	Duration	Intensity*
Recreational	In the past week, did you participate in any non-work recreational activity or exercise, such as walking for exercise, swimming, or dancing that caused at least some increase in breathing and heart rate?	What type of recreational activities did you do during the past week?<br><br>For certain activities: on average, how far did you usually (activity)?	How many times in the past week did you (activity)?	On average, for how many minutes or hours did you usually (activity) at a time?	Thinking about your breathing and heart rate, how hard did this usually feel to you?<br>Fairly light / Somewhat hard / Hard or very hard
Outdoor household activities	In the past week, did you participate in any outdoor household activities such as gardening, mowing, or raking that caused at least some increase in breathing and heart rate?	What type of outdoor household activities did you do during the past week?<br><br>For lifting, carrying, or shoveling: On average, how much did the objects weigh that you (activity)?	How many times in the past week did you (activity)?	On average, for how many minutes or hours did you usually (activity) at a time?	Thinking about your breathing and heart rate, how hard did this usually feel to you?<br>Fairly light / Somewhat hard / Hard or very hard
Indoor household activities	In the past week, did you participate in any indoor household activities such as scrubbing floors, mopping, or vacuuming that caused at least some increase in breathing and heart rate?	What type of indoor household activities did do during the past week?<br><br>For lifting or carrying: On average, how much did the objects weigh that you (activity)?	How many times in the past week did you (activity)?	On average, for how many minutes or hours did you usually (activity) at a time?	Thinking about your breathing and heart rate, how hard did this usually feel to you?<br>Fairly light / Somewhat hard / Hard or very hard
Child and adult care – lifting	Child and adult care activities … would be activities such as playing with children, pushing a stroller or wheelchair, carrying, or lifting a child or adult that you may do in your home or as a volunteer. In the past week, did you participate in any child or adult care activities that caused at least some increase in breathing and heart rate?	What type of child or adult care activities did you do during the past week?<br><br>For lifting or carrying: On average, how much did the objects weigh that you (activity)?	How many times in the past week did you (activity)?	On average, for how many minutes or hours did you usually (activity) at a time?	Thinking about your breathing and heart rate, how hard did this usually feel to you?<br>Fairly light / Somewhat hard / Hard or very hard
Transportation - walk	In the past week, did you walk for transportation, such as to work or to the store, which caused at least some increase in breathing and heart rate?	WALK<br><br>On average, how far did you usually walk one-way?	How many one-way trips did you walk in the past week?	On average, for how many minutes or hours did a one-way walking trip usually take?	Thinking about your breathing and heart rate, how hard did this usually feel to you?<br>Fairly light / Somewhat hard / Hard or very hard
Transportation - bike	In the past week, did you bike for transportation, such as to work or to the store, which caused at least some increase in breathing and heart rate?	BIKE<br><br>On average, how far did you usually bike one-way?	How many one-way trips did you bike in the past week?	On average, for how many minutes or hours did a one-way biking trip usually take?	Thinking about your breathing and heart rate, how hard did this usually feel to you?<br>Fairly light / Somewhat hard / Hard or very hard
Work and school	In the past week, did you participate in any work activities such as walking,	What type of work activities did you do during the past	How many times in the past week did you	On average, for how many minutes or hours did you	Thinking about your breathing and heart rate, how hard did

activities	lifting, or carrying objects, that caused at least some increase in breathing and heart rate?	week?<br><br>For carrying or shoveling: On average, how much did the objects weigh that you (activity)?	(activity)?<br><br>For walking: On average, how far did you usually walk?	usually (activity) at a time?	this usually feel to you? Fairly light / Somewhat hard / Hard or very hard
Other activity	Before we move on to another section, I want to be sure you had a chance to tell me about all the activities you did in the past week that caused at least some increase in breathing and heart rate. Can you think of any other activities, including lifting, you did in the past week that we have not talked about?	What other activities did you do during the past week?<br><br>For some activities: On average, how far did you usually (activity)?	How many times in the past week did you (activity) at a time?<br><br>For lifting, carrying, or shoveling: On average, how much did the objects weigh that you (activity)?	On average, for how many minutes or hours did you usually (activity) at a time?	Thinking about your breathing and heart rate, how hard did this usually feel to you? Fairly light / Somewhat hard / Hard or very hard

Note: The lead in question described the questionnaire in this way: "Now I am going to ask you some questions about physical activities you might do at work, at home, for recreation, and about activities involving child or adult care. I want you to tell me about activities you did that "caused at least some increase in breathing and heart rate". The questions ask about the past week, meaning the last 7 days not including today, so that would mean from last <*day*> to yesterday or <*day*>."

*Intensity was defined as not hard = did not feel any increase in breathing or heart rate and thus not recorded; fairly light = at least some increase in breathing and heart rate; somewhat hard = moderate increase in breathing and heart rate; and hard or very hard = large increase in breathing and heart rate.
