# Supplementary material for: Measuring physical activity among pregnant women using a structured one-week recall questionnaire: evidence for validity and reliability
Source: Int J Behav Nutr Phys Act. 2010 Mar 21;7:21. doi: 10.1186/1479-5868-7-21 (PMC2855515; doi:10.1186/1479-5868-7-21)
Supplement: Additional file 2 — PIN3 physical activity diary card. This file provides the diary card used for the validation component in this study, along with a scoring table. [file 1479-5868-7-21-S2.PDF]

PIN3 Physical Activity Diary Card

Patid_______________ Labid_____________

Write in Date: ____/____/____ ____/____/____ ____/____/____ ____/____/____ ____/____/____ ____/____/____ ____/____/____

	**Day 1**	**Day 2**	**Day 3**	**Day 4**	**Day 5**	**Day 6**	**Day 7**
**Outdoor Activity**<br>Gardening	_____hrs ____mins<br>FL SH H	_____hrs ____mins<br>FL SH H	_____hrs ____mins<br>FL SH H	_____hrs ____mins<br>FL SH H	_____hrs ____mins<br>FL SH H	_____hrs ____mins<br>FL SH H	_____hrs ____mins<br>FL SH H
Mowing	_____hrs ____mins<br>FL SH H	_____hrs ____mins<br>FL SH H	_____hrs ____mins<br>FL SH H	_____hrs ____mins<br>FL SH H	_____hrs ____mins<br>FL SH H	_____hrs ____mins<br>FL SH H	_____hrs ____mins<br>FL SH H
Raking	_____hrs ____mins<br>FL SH H	_____hrs ____mins<br>FL SH H	_____hrs ____mins<br>FL SH H	_____hrs ____mins<br>FL SH H	_____hrs ____mins<br>FL SH H	_____hrs ____mins<br>FL SH H	_____hrs ____mins<br>FL SH H
**Indoor Activity**<br>Scrubbing floors	_____hrs ____mins<br>FL SH H	_____hrs ____mins<br>FL SH H	_____hrs ____mins<br>FL SH H	_____hrs ____mins<br>FL SH H	_____hrs ____mins<br>FL SH H	_____hrs ____mins<br>FL SH H	_____hrs ____mins<br>FL SH H
Mopping	_____hrs ____mins<br>FL SH H	_____hrs ____mins<br>FL SH H	_____hrs ____mins<br>FL SH H	_____hrs ____mins<br>FL SH H	_____hrs ____mins<br>FL SH H	_____hrs ____mins<br>FL SH H	_____hrs ____mins<br>FL SH H
Sweeping with a broom	_____hrs ____mins<br>FL SH H	_____hrs ____mins<br>FL SH H	_____hrs ____mins<br>FL SH H	_____hrs ____mins<br>FL SH H	_____hrs ____mins<br>FL SH H	_____hrs ____mins<br>FL SH H	_____hrs ____mins<br>FL SH H
Laundry	_____hrs ____mins<br>FL SH H	_____hrs ____mins<br>FL SH H	_____hrs ____mins<br>FL SH H	_____hrs ____mins<br>FL SH H	_____hrs ____mins<br>FL SH H	_____hrs ____mins<br>FL SH H	_____hrs ____mins<br>FL SH H
Vacuuming	_____hrs ____mins<br>FL SH H	_____hrs ____mins<br>FL SH H	_____hrs ____mins<br>FL SH H	_____hrs ____mins<br>FL SH H	_____hrs ____mins<br>FL SH H	_____hrs ____mins<br>FL SH H	_____hrs ____mins<br>FL SH H
**Working activity**<br>Walking at work	_____hrs ____mins<br>FL SH H	_____hrs ____mins<br>FL SH H	_____hrs ____mins<br>FL SH H	_____hrs ____mins<br>FL SH H	_____hrs ____mins<br>FL SH H	_____hrs ____mins<br>FL SH H	_____hrs ____mins<br>FL SH H
Lifting at work	_____hrs ____mins<br>FL SH H	_____hrs ____mins<br>FL SH H	_____hrs ____mins<br>FL SH H	_____hrs ____mins<br>FL SH H	_____hrs ____mins<br>FL SH H	_____hrs ____mins<br>FL SH H	_____hrs ____mins<br>FL SH H
Carrying objects at work	_____hrs ____mins<br>FL SH H	_____hrs ____mins<br>FL SH H	_____hrs ____mins<br>FL SH H	_____hrs ____mins<br>FL SH H	_____hrs ____mins<br>FL SH H	_____hrs ____mins<br>FL SH H	_____hrs ____mins<br>FL SH H
**Transportation**<br>Walking to and from for transportation	_____hrs ____mins<br>FL SH H	_____hrs ____mins<br>FL SH H	_____hrs ____mins<br>FL SH H	_____hrs ____mins<br>FL SH H	_____hrs ____mins<br>FL SH H	_____hrs ____mins<br>FL SH H	_____hrs ____mins<br>FL SH H
Cycling to and from for transportation	_____hrs ____mins<br>FL SH H	_____hrs ____mins<br>FL SH H	_____hrs ____mins<br>FL SH H	_____hrs ____mins<br>FL SH H	_____hrs ____mins<br>FL SH H	_____hrs ____mins<br>FL SH H	_____hrs ____mins<br>FL SH H

FL - Fairly Light SH - Somewhat Hard H - Hard or Very Hard **(over for more activity choices)**

Continuation of PIN Physical Activity Diary Card

	Day 1	Day 2	Day 3	Day 4	Day 5	Day 6	Day 7
**Recreational Activities**<br>Walking for exercise	_____hrs ____mins<br>FL SH H	_____hrs ____mins<br>FL SH H	_____hrs ____mins<br>FL SH H	_____hrs ____mins<br>FL SH H	_____hrs ____mins<br>FL SH H	_____hrs ____mins<br>FL SH H	_____hrs ____mins<br>FL SH H
Swimming	_____hrs ____mins<br>FL SH H	_____hrs ____mins<br>FL SH H	_____hrs ____mins<br>FL SH H	_____hrs ____mins<br>FL SH H	_____hrs ____mins<br>FL SH H	_____hrs ____mins<br>FL SH H	_____hrs ____mins<br>FL SH H
Jogging	_____hrs ____mins<br>FL SH H	_____hrs ____mins<br>FL SH H	_____hrs ____mins<br>FL SH H	_____hrs ____mins<br>FL SH H	_____hrs ____mins<br>FL SH H	_____hrs ____mins<br>FL SH H	_____hrs ____mins<br>FL SH H
Bicycling	_____hrs ____mins<br>FL SH H	_____hrs ____mins<br>FL SH H	_____hrs ____mins<br>FL SH H	_____hrs ____mins<br>FL SH H	_____hrs ____mins<br>FL SH H	_____hrs ____mins<br>FL SH H	_____hrs ____mins<br>FL SH H
Circuit training/ weight lifting/conditioning	_____hrs ____mins<br>FL SH H	_____hrs ____mins<br>FL SH H	_____hrs ____mins<br>FL SH H	_____hrs ____mins<br>FL SH H	_____hrs ____mins<br>FL SH H	_____hrs ____mins<br>FL SH H	_____hrs ____mins<br>FL SH H
**Child/Adult Care**<br>Playing w/ children	_____hrs ____mins<br>FL SH H	_____hrs ____mins<br>FL SH H	_____hrs ____mins<br>FL SH H	_____hrs ____mins<br>FL SH H	_____hrs ____mins<br>FL SH H	_____hrs ____mins<br>FL SH H	_____hrs ____mins<br>FL SH H
Pushing stroller/wheelchair	_____hrs ____mins<br>FL SH H	_____hrs ____mins<br>FL SH H	_____hrs ____mins<br>FL SH H	_____hrs ____mins<br>FL SH H	_____hrs ____mins<br>FL SH H	_____hrs ____mins<br>FL SH H	_____hrs ____mins<br>FL SH H
Carrying/lifting child or adult	_____hrs ____mins<br>FL SH H	_____hrs ____mins<br>FL SH H	_____hrs ____mins<br>FL SH H	_____hrs ____mins<br>FL SH H	_____hrs ____mins<br>FL SH H	_____hrs ____mins<br>FL SH H	_____hrs ____mins<br>FL SH H
**Miscellaneous**<br>Climbing stairs	_____hrs ____mins<br>FL SH H	_____hrs ____mins<br>FL SH H	_____hrs ____mins<br>FL SH H	_____hrs ____mins<br>FL SH H	_____hrs ____mins<br>FL SH H	_____hrs ____mins<br>FL SH H	_____hrs ____mins<br>FL SH H
**Other Activities**<br>(please list)	_____hrs ____mins<br>FL SH H	_____hrs ____mins<br>FL SH H	_____hrs ____mins<br>FL SH H	_____hrs ____mins<br>FL SH H	_____hrs ____mins<br>FL SH H	_____hrs ____mins<br>FL SH H	_____hrs ____mins<br>FL SH H
	_____hrs ____mins<br>FL SH H	_____hrs ____mins<br>FL SH H	_____hrs ____mins<br>FL SH H	_____hrs ____mins<br>FL SH H	_____hrs ____mins<br>FL SH H	_____hrs ____mins<br>FL SH H	_____hrs ____mins<br>FL SH H
	_____hrs ____mins<br>FL SH H	_____hrs ____mins<br>FL SH H	_____hrs ____mins<br>FL SH H	_____hrs ____mins<br>FL SH H	_____hrs ____mins<br>FL SH H	_____hrs ____mins<br>FL SH H	_____hrs ____mins<br>FL SH H

FL - Fairly Light SH - Somewhat Hard H - Hard or Very Hard

Fill out the time and date you stop wearing the monitor: TIME: ____:_____ am pm (circle one) DATE:___ ___/___ ___/___ ___

Instructions

1. Begin recording your physical activity on this card the day after you receive the activity monitor.
   Example: You received the monitor at the clinic on Monday you start wearing it then. On Tuesday morning you put the monitor on when getting dressed, and this becomes your first day of recording your activities for the diary card. So, Tuesday evening you would begin to fill out the diary card about your activities and ***Tuesday as Day 1 on the diary card.***

2. Please **fill out the diary card at the end of every day** and do not wait until the end of the week to record all activities.

3. For each activity you perform, please write in the estimated number of hours or minutes you did the activity that day.

4. For each activity circle the appropriate difficulty level of the activity:

   FL - Fairly Light = at least some increase in breathing and heart rate
   SH - Somewhat Hard = moderate increase in breathing and heart rate
   H - Hard or Very Hard = large increase in breathing and heart rate

5. If you did not perform an activity, then please leave that block blank.

6. If you performed an activity that is not listed, then add it to the "other activities" section on the bottom of the backside of the diary card.

7. Only record activities that caused at least **some increase in breathing or heart rate.**

8. **Return this card with the monitor in the envelope provided to you the day after you stop wearing the monitor.**

9. **Fill out the time and date you <u>stop wearing</u> the monitor in the space provided at the bottom of diary card.**

10. **If you forget to wear the monitor, put it on as soon as possible!**

Table: Assignment of compendium codes and metabolic equivalent (MET) values to the activities on the PIN3 Physical Activity Diary Card

Activities From the Diary Card	Code*	MET Value
OUTDOOR ACTIVITY		
Gardening	08245	4.0
Mowing	08095	5.5
Raking	08160	4.3
INDOOR ACTIVITY		
Scrubbing floors	05131	3.8
Mopping	05021	3.5
Sweeping with a broom	05010	3.3
Laundry	05090	1.5
Vacuuming	05043	3.5
WORK		
Walking at work	11794	3.3
Lifting at work	11631	4.0
Carrying objects at work	11051	4.0
TRANSPORTATION		
Walking to and from for transportation	17270	4.0
Cycling to and from for transportation	01010	4.0
RECREATIONAL ACTIVITIES		
Walking for exercise	17153	3.7
Swimming	18225	7.0
Jogging	12020	7.0
Bicycling	01015	8.0
Circuit training/ weight lifting/conditioning	02130	3.0
CHILD ADULT CARE		
Playing with children	05172	3.4
Pushing stroller/wheelchair	17100	2.5
Carrying/lifting child or adult	05181	3.0
Climbing stairs	17130	8.0
Other Activities*		

*Other activities were assigned codes and corresponding MET values according to the compendium (Ainsworth et al 2000). The final compendium of activities used for scoring is available at http://www.cpc.unc.edu/projects/pin/design_pin3/docs_3/PIN-MET-Table-080207.pdf.
