# Supplementary figures and images for: Measuring physical activity among pregnant women using a structured one-week recall questionnaire: evidence for validity and reliability
Source: Int J Behav Nutr Phys Act. 2010 Mar 21;7:21. doi: 10.1186/1479-5868-7-21 (PMC2855515; doi:10.1186/1479-5868-7-21)

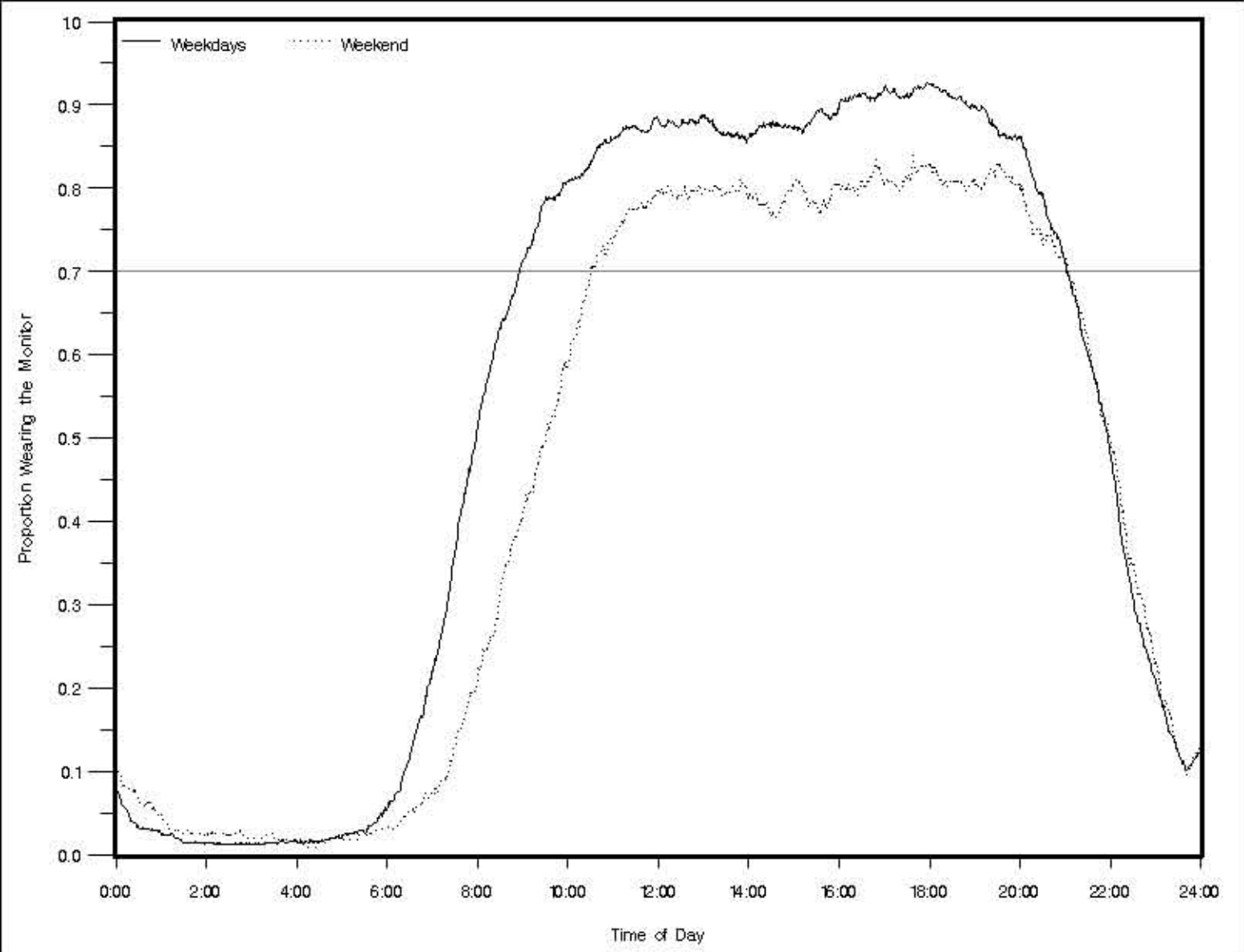

Supplement: Additional file 3 — Cumulative proportion of the validity participants (n = 177) wearing the accelerometer by time of day, separately for weekdays and weekends. This file provides a summary of the accelerometer wearing time among the pregnant women who participated in the validity study. [file 1479-5868-7-21-S3.PDF]
