# Supplementary material for: Measuring physical activity among pregnant women using a structured one-week recall questionnaire: evidence for validity and reliability
Source: Int J Behav Nutr Phys Act. 2010 Mar 21;7:21. doi: 10.1186/1479-5868-7-21 (PMC2855515; doi:10.1186/1479-5868-7-21)
Supplement: Additional file 4 — Physical activity for the validity and reliability study participants. This file provides a summary of the physical activity among pregnant women who participated in the validity or reliability study. [file 1479-5868-7-21-S4.PDF]

Table: Physical activity for the validity and reliability study participants

	Validity Study						Reliability Study**		
	Complete Actigraph Sample* (n=120)			Full Sample (n=177)			n=109		
	Mean	Median	IQ range	Mean	Median	IQ range	Mean	Median	IQ range
**Questionnaire**									
Using perceived intensity (hours/week)									
Moderate activity	3.8	2.2	0.8-5.0	3.8	1.8	0.5-4.9	3.5	1.0	0.0-3.5
Vigorous activity	0.7	0.0	0.0-0.3	0.6	0.0	0.0-0.2	0.3	0.0	0.0-0.0
Moderate to vigorous activity	4.5	2.8	0.9-5.6	4.4	2.3	0.8-5.3	3.8	1.3	0.0-4.3
Total physical activity	10.3	7.1	4.1-13.0	10.5	7.1	4.1-12.3	8.7	5.3	2.1-10.3
Using absolute intensity (MET-hours/week)									
Total physical activity	35.5	27.2	16.2-43.9	36.3	26.1	15.8-43.7	30.1	18.1	7.3-39.0
Moderate intensity defined as 3-6 METS									
Moderate activity	27.9	17.1	9.4-32.7	29.4	17.3	10.1-31.6	24.3	13.6	4.0-29.1
Vigorous activity	3.0	0.0	0.0-3.1	2.6	0.0	0.0-1.3	2.8	0.0	0.0-0.0
Moderate to vigorous activity	30.9	21.5	13.8-35.5	32.0	21.3	13.0-36.2	27.1	15.8	6.3-34.8
Moderate intensity defined as 4.8-7.1 METS									
Moderate activity	3.6	0.0	0.0-3.1	3.6	0.0	0.0-3.0	2.7	0.0	0.0-0.0
Vigorous activity	1.6	0.0	0.0-0.0	1.4	0.0	0.0-0.0	1.5	0.0	0.0-0.0
Moderate to vigorous activity	5.2	0.8	0.0-7.0	5.0	0.0	0.0-6.7	4.2	0.0	0.0-2.8
**Diary**									
Using perceived intensity (hours/week)									
Moderate activity	6.9	3.5	1.5-8.1	8.0	3.5	1.5-9.8			
Vigorous activity	0.8	0.0	0.0-0.4	0.8	0.0	0.0-0.4			
Moderate to vigorous activity	7.6	3.9	1.8-8.3	8.8	3.8	1.8-10.0			
Total physical activity	20.0	13.4	6.5-23.9	22.0	14.6	6.8-27.8			
Using absolute intensity (MET-hours/week)									
Total physical activity	68.5	45.3	24.3-83.9	75.3	47.2	25.7-92.9			

<u>Moderate intensity defined as 3-6 METS</u>						
Moderate activity	57.5	38.5	16.3-69.6	64.9	40.5	18.5-78.0
Vigorous activity	5.2	0.0	0.0-4.2	4.7	0.0	0.0-3.5
Moderate to vigorous activity	62.7	41.3	20.7-72.8	69.6	43.1	23.4-81.6
<u>Moderate intensity defined as 4.8-7.1 METS</u>						
Moderate activity	5.0	0.0	0.0-5.4	4.6	0.0	0.0-4.7
Vigorous activity	1.5	0.0	0.0-0.0	1.3	0.0	0.0-0.0
Moderate to vigorous activity	6.5	0.2	0.0-7.0	5.9	0.0	0.0-7.0
**<u>Accelerometry</u>**						
<u>Freedson et al (hours/week)</u>						
Moderate activity	2.5	2.2	1.3-3.6	2.1	1.8	0.9-3.1
Vigorous activity	0.3	0.0	0.0-0.1	0.2	0.0	0.0-0.1
Moderate to vigorous activity	2.7	2.4	1.3-4.0	2.4	1.8	0.9-3.6
<u>Swartz et al (hours/week)</u>						
Moderate activity	15.3	14.4	11.3-17.9	13.5	12.2	9.6-16.5
Vigorous activity	0.4	0.0	0.0-0.4	0.3	0.0	0.0-0.2
Moderate to vigorous activity	15.7	15.3	11.5-18.0	13.8	13.0	9.7-17.0
<u>Troiano et al (hours/week)</u>						
Moderate activity	2.4	2.1	1.2-3.4	2.0	1.6	0.8-3.1
Vigorous activity	0.2	0.0	0.0-0.1	0.2	0.0	0.0-0.0
Moderate to vigorous activity	2.6	2.2	1.2-3.9	2.2	1.7	0.8-3.4

*Complete Actigraph data (n=120) was defined as having nonmissing counts over at least 70% of a standard measurement day, with a standard measurement day defined as the length of time in which at least 70% of participants were wearing the monitor. This was defined separately for weekdays and weekends.

**The reliability study participants did not complete the diary or wear the accelerometer. The data presented are from time 1.

The cutpoints on accelerometer counts per minute were defined as follows:

Freedson et al: 1952-5724 (Moderate); >=5725 (Vigorous)
Swartz et al: 574-4944 (Moderate); >=4945 (Vigorous)
Troiano et al: 2020-5998 (Moderate); >=5999 (Vigorous)
